# TQFL12, a novel synthetic derivative of TQ, inhibits triple‐negative breast cancer metastasis and invasion through activating AMPK/ACC pathway

**DOI:** 10.1111/jcmm.16945

**Published:** 2021-10-05

**Authors:** Chunli Wei, Hui Zou, Ting Xiao, Xiaoyan Liu, Qianqian Wang, Jingliang Cheng, Shangyi Fu, Jiangzhou Peng, Xin Xie, Junjiang Fu

**Affiliations:** ^1^ Key Laboratory of Epigenetics and Oncology the Research Center for Preclinical Medicine Southwest Medical University Luzhou China; ^2^ Key Laboratory of Study and Discovery of Small Targeted Molecules of Hunan Province School of Medicine Hunan Normal University Changsha China; ^3^ Chronic Disease Research Center Medical College Dalian University Dalian China; ^4^ Human Genome Sequencing Center Baylor College of Medicine Houston TX USA; ^5^ School of Medicine Baylor College of Medicine Houston TX USA; ^6^ Department of Thoracic Surgery The Third Affiliated Hospital of Southern Medical University Guangzhou China; ^7^ Department of Orthopedics The Second Affiliated Hospital of Zhejiang University School of Medicine Zhejiang University Hangzhou China; ^8^ Zhejiang University‐University of Edinburgh Institute Zhejiang University School of Medicine Zhejiang University Hangzhou China; ^9^ Dr. Li Dak Sum & Yip Yio Chin Center for Stem Cell and Regenerative Medicine Zhejiang University Hangzhou China

**Keywords:** AMPK, anti‐cancer, metastasis, thymoquinone derivative, triple‐negative breast cancer

## Abstract

Thymoquinone (TQ) has been reported as an anti‐tumour drug widely studied in various tumours, and its mechanism and effect of which has become a focus of current research. However, previous studies from our laboratory and other groups found that TQ showed weak anti‐tumour effects in many cancer cell lines and animal models. Therefore, it is necessary to modify and optimize the structure of TQ to obtain new chemical entities with high efficiency and low toxicity as candidates for development of new drugs in treating cancer. Therefore, we designed and synthesized several TQ derivatives. Systematic analysis, including *in vitro* and *in vivo*, was conducted on a panel of triple‐negative breast cancer (TNBC) cells and mouse model to demonstrate whether TQFL12, a new TQ derivative, is more efficient than TQ. We found that the anti‐proliferative effect of TQFL12 against TNBC cells is significantly stronger than TQ. We also demonstrated TQFL12 affects different aspects in breast cancer development including cell proliferation, migration, invasion and apoptosis. Moreover, TQFL12 inhibited tumour growth and metastasis in cancer cell–derived xenograft mouse model, with less toxicity compared with TQ. Finally, mechanism research indicated that TQFL12 increased AMPK/ACC activity by stabilizing AMPKα, while molecular docking supported the direct interaction between TQFL12 and AMPKα. Taken together, our findings suggest that TQFL12, as a novel chemical entity, possesses a better inhibitory effect on TNBC cells and less toxicity in both *in vitro* and *in vivo* studies. As such, TQFL12 could serve as a potential therapeutic agent for breast cancer.

## 1 INTRODUCTION

Malignant tumours are the main cause of death of cancer patients, and the mechanism and therapeutics of tumour metastasis has become the most widely researched topic recently. Breast cancer is a worldwide malignant disease that seriously threatens mental and physical health of patients.^1^, ^2^, ^3^, ^4^ Triple‐negative breast cancer (TNBC) and metastatic breast cancer (mBC) are aggressive and highly heterogeneous subtypes of breast cancer with poor prognosis, which continue to be a leading cause of cancer‐related death in women.^4^, ^5^ Even though mortality rates have reduced over the recent years, the 5‐year survival rate of advanced TNBC is still very low.^6^, ^7^ Scientists are attempting to tackle this challenge and to develop new therapies for those breast cancer patients. Among various tumour treatment options, targeting therapy using small molecule drugs is so far the most effective treatment strategy.^8^

Bioactive agents derived from natural products have gained substantial attentions and have long been used as therapeutic drug owing to their anti‐cancer, anti‐inflammatory, neuroprotective and other properties.^9^, ^10^, ^11^ One of the most successful natural agents is artemisinin (Qinghaosu), which is considered as a gift in old Chinese medicine discovered by Youyou Tu.^12^ However, lots of compounds from traditional Chinese medicine (TCM) have not been fully accepted, mainly due to their limited effects, poorly defined molecular mechanisms and high costs.

Moreover, malignant tumours are prone to developing resistance or inducing side effects in the host in response to traditional chemotherapy. Thus, finding a natural drug that is highly effective with low toxicity has become an urgent issue. Interestingly, thymoquinone (TQ), the main active compound isolated from black seed oil (Nigella sativa), has been reported to be a potential treatment option for a variety of diseases including cancer.^13^, ^14^ TQ can inhibit cancer cell metastases in various malignant tumours including prostate cancer,^15^ breast cancer,^16^, ^17^ bladder cancer^18^ and lung cancer.^19^, ^20^ With these promising therapeutic effects, TQ has a potential to being a new clinical treatment option.^21^, ^22^, ^23^

While it does have the prospect, there are limitations in using TQ with its original form. For instance, the effective drug concentration of TQ is reportedly high and the latest studies reported that the IC_50_ of TQ is higher than165µM in some breast cancer cells.^24^ On the other side, derivatives of TQ were rarely studied, raising the research question of whether TQ derivatives could be new avenues for cancer treatment. As such, additional studies are necessary to assay TQ derivatives for their efficacy, toxicity and anti‐tumour properties. In this study, we obtained new chemical entities via chemical modification and improved the bioactivity of TQ. A new compound, (*E*)‐3‐((4‐chlorobenzylidene)amino)‐5‐isopropyl‐2‐methylcyclohexa‐2, 5‐diene‐1,4‐dione (TQFL12, molecular formula: C_17_H_16_ClNO_2_), was synthesized and its in *vitro* and in *vivo* anti‐tumour activities against triple‐negative breast cancer (TNBC) were evaluated. Moreover, we also investigated its molecular mechanism.

## 2 MATERIALS AND METHODS

### 2.1 Reagents and cell culture

TQ and Corning Matrigel Matrix GFR were purchased from Sigma‐Aldrich. CCK8 reagent was purchased from Shanghai Dojindo Chemical Technology Co., Ltd. Foetal bovine serum (FBS) was purchased from PAN‐Biotech. DMEM and RPMI 1640medium were obtained from Gibco. MCF‐10A cell special medium was purchased from Saizhe Biological Technology Co., Ltd. Antibiotics (penicillin‐streptomycin), trypsin‐EDTA and 4% paraformaldehyde were purchased from Beyotime Biological Technology Co., Ltd. Annexin V/Propidium iodide (PI) staining kit was purchased from BD Biosciences. The antibodies against phospho‐AMPKα (Thr172, 1:3000, 2535; Cell Signaling), AMPKα (1:3000, 2532; Cell Signaling), phospho‐ACC (S79, 1:2000; 530298, ENBIO), Actin (1:5000, A1978; Sigma), the anti‐rabbit secondary antibodies (1:5000, 7074; Cell Signaling), the anti‐mouse secondary antibodies (1:5000, 7076; Cell Signaling). Schrodinger software of molecular docking was obtained from Schrodinger Inc (New York, NY, 2009). Cycloheximide was obtained from Sigma. The BALB/c mice were purchased from Tengxin Biotechnology Co., Ltd.

All breast cancer cell lines (BT549, MDA‐MB‐231, 4T1) and normal mammary epithelial cell line (MCF‐10A) were obtained from ATCC (American Type Culture Collection) and were maintained at 37°C with 5% CO_2_ in medium supplemented with 10% FBS.

### 2.2 Chemicals

NaN_3_ (DDN) was bought from Xiya Reagent. 4‐Chlorobenzaldehyde was from Energy Chemical. Hydrochloric acid (HCl, analytical grade) was purchased from Zhuzhou Quartz Glass Co. LTD, and anhydrous ethyl alcohol (EtOH) was from Sinopharm Group Chemical Reagent Co. LTD (Shanghai, China). ^1^H and ^13^C NMR experiments were recorded on a Bruker AV‐400 MHz spectrometer (Bruker) with tetramethylsilane (TMS) as the internal standard when DMSO‐*d*_6_ was used as a solvent. Chemical shifts are expressed in *δ* (ppm) and coupling constants (*J*) in Hz. High‐resolution (ESI) MS spectra were recorded using an Agilent 6550 iFunnel Q‐TOF LC/MS system. HPLC analysis was used to determine the purity (>98%) of the compound with a YMC Pack ODS‐A (5 μm, 250 × 4.6 mm; YMC Co. Ltd) column.

### 2.3 Cell Counting Kit‐8 assays

Cells were plated in a 96‐well plate with 3000–5000 cells/well and treated with various concentrations of TQ or TQFL12 for 16, 24 or 48 h. After the treatment, 10 µl of CCK8 reagent was added to each well and incubated at 37°C for 1 h. After incubation, the absorbance at 450 nm was measured using microplate reader.^25^ Each experiment was repeated three times.

### 2.4 Cell growth, migration and invasion assays

Cell suspensions with 100 µl (1 × 10^4^ cells/ml) were placed on each of the 16‐well E‐plate for cell growth. For cell migration and invasion index analysis, CMI plates were used, and the lower chamber wells were filled with chemotaxis inducer (10% serum‐supplemented media), and upper chamber contained additional cell suspensions (1 × 10^4^ cells/ml). For cell invasion assay, Matrigel with 1:40 diluted in 1 × PBS was seeded on the CMI plate before assay. After 8h of cell growth, 2.5 µM or 5 µM of TQFL12 or DMSO was added. The cell migration and invasion process were detected every 30 min by a real‐time cell analyser (xCELLigence RTCA DP; Roche, Germany).^26^, ^27^, ^28^ Each experiment was repeated three times.

### 2.5 Apoptosis and cell cycle assays

Apoptosis and cell cycle assays were performed as previously described.^25^ 4T1 or MDA‐MB‐231 cells (1.5 × 10^5^ cells/well) were seeded in 6‐well plate and treated with different concentrations (0, 2.5, 5 μM) of TQFL12 for another 24 h. The cells were stained with Annexin‐V FITC and PI in the dark. The apoptotic cells were analysed by flow cytometer. For cell cycle analysis, the cells were stained in 300 μl PI solution in dark Then, each stage of cell cycles was analysed by flow cytometer.^25^ Each experiment was repeated three times.

### 2.6 Mouse xenograft assays

Animal experiments of mice were in compliance with institutional animal care guidelines and followed the university committee–approved protocols.^16^

To establish the breast cancer xenograft model, cells of mouse triple‐negative breast cancer (4T1) were injected into the mammary fat pads of female BALB/c mice and the size of the tumours were measured every 5 days.^26^ Four days after injection of the cells, the mice were randomly divided into seven groups with six in each group and treated with 0, 3.75 mg/kg, 7.5 mg/kg and 15 mg/kg of TQFL12, with 0, 3.75 mg/kg, 7.5 mg/kg and 15 mg/kg of TQ. The tumour sizes were continuously monitored during the treatment. At the end of the 27‐day treatment (30‐day of 4T1 cell injection), the mice were killed, the tumour tissues were dissected, and the weight of the tumour tissues was measured. To estimate the TQFL12 and TQ effects on tumour cell migration/invasion, the lungs of the animals were dissected out at the end of the treatment and the number of colonies formed in the lungs was counted.

### 2.7 Histology

Tumour tissues were fixed in 4% paraformaldehyde for 24 h, embedded in paraffin and sliced in 5 µm thickness. After dewaxing in a xylene series, the slides were dehydrated in alcohol and stained with H&E (haematoxylin and eosin).^26^

### 2.8 Western blotting and RT‐PCR

4T1, MDA‐MB‐231 or BT549 cells were seeded in 6‐well plate and were incubated for 24 h, then treated with different concentrations (0, 2.5, 5 μM) of TQFL12 and lysed by adding lysis buffer. Cell lysates with 40μg of proteins were separated on 8%, 10% or 12% SDS‐PAGE and transferred to the nitrocellulose membrane. After blocking, the membrane was incubated with primary antibodies and secondary antibodies. The intensity of each band on the membrane was detected by imaging scanner.

BT549 cells were seeded in 6‐well plate and incubated for 24 h and then treated with different concentrations (0, 2.5, 5, 10 μM) of TQFL12 for 12 h. Total RNA was extracted and reverse‐transcribed into cDNA. Semi‐quantitative RT‐PCR for the *PRKAA1* gene was performed using primers (forward primer: 5’‐ggagccttgatgtggtagga‐3’, reverse primer: 5’‐tttcatccagccttccattc‐3’). *GAPDH* was served as internal control. Each experiment was repeated three times.

### 2.9 Assays for the treatments of cycloheximide and TQFL12

4T1 or BT549 cells were treated with or without TQFL12 (5 μM) and were cultured for 24 h before 0.1 mg/ml (20 µM) of cycloheximide (CHX) was added. Cells were harvested and proteins in the lysate were used for Western blotting. Band intensities were semi‐quantified by densitometry and analysed using imaging scanner.

### 2.10 Molecular docking

The file of AMPKα (NO.4CFH) from PDB (Protein Data Bank) was selected, and docking was conducted by Glide program in Schrodinger software.^25^ TQFL12 was docked into the binding site of the AMPKα with the standard precision scoring mode.^25^

### 2.11 Statistical analysis

Three individual experiments were performed, and all data are presented as the mean ± standard deviation SD. The statistical differences were performed by one‐way ANOVA using GraphPad Prism 6. *p* value <0.05 was considered significantly different. *p < 0.05, 0.01< **p < 0.05, ***p < 0.01, ****p < 0.001 are indicated as differences with *p* values.

## 3 RESULTS

### 3.1 Design and synthesis of TQFL12, a novel TQ derivative

**FIGURE 1:**
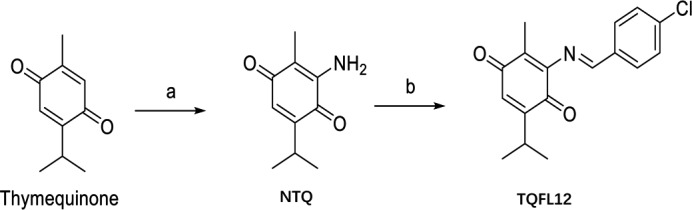
The synthesis and structure of TQFL12. Synthetic route of TQFL12: (a) NaN_3_, CH_3_COOH, EtOH, reflux, 6 h; (b) HCl, EtOH, reflux, 8 h. NTQ: 3‐amino‐5‐isopropyl‐2‐methylcyclohexa‐2,5‐diene‐1,4‐dione. TQFL12: (*E*)‐3‐((4‐chlorobenzylidene) amino)‐5‐isopropyl‐2‐methylcyclohexa‐2,5‐diene‐1,4‐dione. Molecular formula for TQFL12 is C17H16ClNO2. h, hours

With TQ as the starting compound, we decided to add different chemically groups to TQ. Two steps were used to synthesize TQFL12 (molecular formula: C_17_H_16_ClNO_2_). First, synthesis of 3‐amino‐5‐isopropyl‐2‐methylcyclohexa‐2,5‐diene‐1,4‐dione (NTQ): A solution of TQ (1.640 g, 10 mmol) was performed by dissolving anhydrous NaN_3_ in anhydrous EtOH (80 ml) and then adding acetic acid (30 ml). After heating and stirring at 80°C for 6 h, the reaction mixture was cooled to room temperature (RT). TLC verified that TQ had been reacted completely. The reaction mixture was run on a silica gel to obtain the pure product (NTQ) (Figure 1). NTQ shows red solid, yield 1.16 g (65%); ^1^H NMR (400 MHz, DMSO‐*d*_6_) (Figure S1) *δ*: 1.05 (6H, d, *J* = 6.8 Hz), 1.73 (3H, s), 2.87 (1H, m), 6.28 (1H, s), 6.48 (2H, 2) ^13^C NMR (100 MHz, DMSO‐*d*_6_) (Figure S2): 9.11, 21.6, 26.3, 106.8, 132.5, 145.7, 149.5, 184.1, 185.3.

Then, further synthesis of (*E*)‐3‐((4‐chlorobenzylidene) amino)‐5‐isopropyl‐2‐methylcyclohexa‐2,5‐diene‐1,4‐dione (TQFL12) involved a mixture of compound NTQ (0.179 g, 1 mmol) and 4‐chlorobenzaldehyde (0.144, 1 mmol) in EtOH (20 ml) with HCl (0.5 ml) heated and stirred at 80 °C for 8 h. The resulting mixture was filtered to obtain the filtrate. The filtrate was concentrated under reduced pressure to get crude product and recrystallized from ethanol to give the pure compound TQFL12 (Figure 1). TQFL12 was a pale yellow solid and yielded 255 mg (85.1%%); ^1^H NMR (400 MHz, DMSO‐*d*_6_) (Figure S3) *δ*: 1.33 (6H, d), 2.33 (3H, s), 3.24 (1H, m), 6.77 (1H, d), 7.62 (2H, m), 8.14 (2H, m), 9.22 (1H, s); ^13^C NMR (Figure S4) (100 MHz, DMSO‐*d*_6_): 10.6, 22.9, 29.3, 111.3, 112.2, 126.3, 128.7, 129.1, 129.8, 136.5, 142.1, 142.2, 152.8, 161.0; HR‐ESI‐MS (Figure S5): *m*/*z* 302.0949 [M+H]^+^ (calcd. for C_17_H_17_ClNO_2_^+^: 302.0948). The purity of TQFL12 is 98.2% (Figure S6).

### 3.2 The cytotoxic sensitivity of TQFL12 is higher than TQ on different breast cancer cells

**FIGURE 2:**
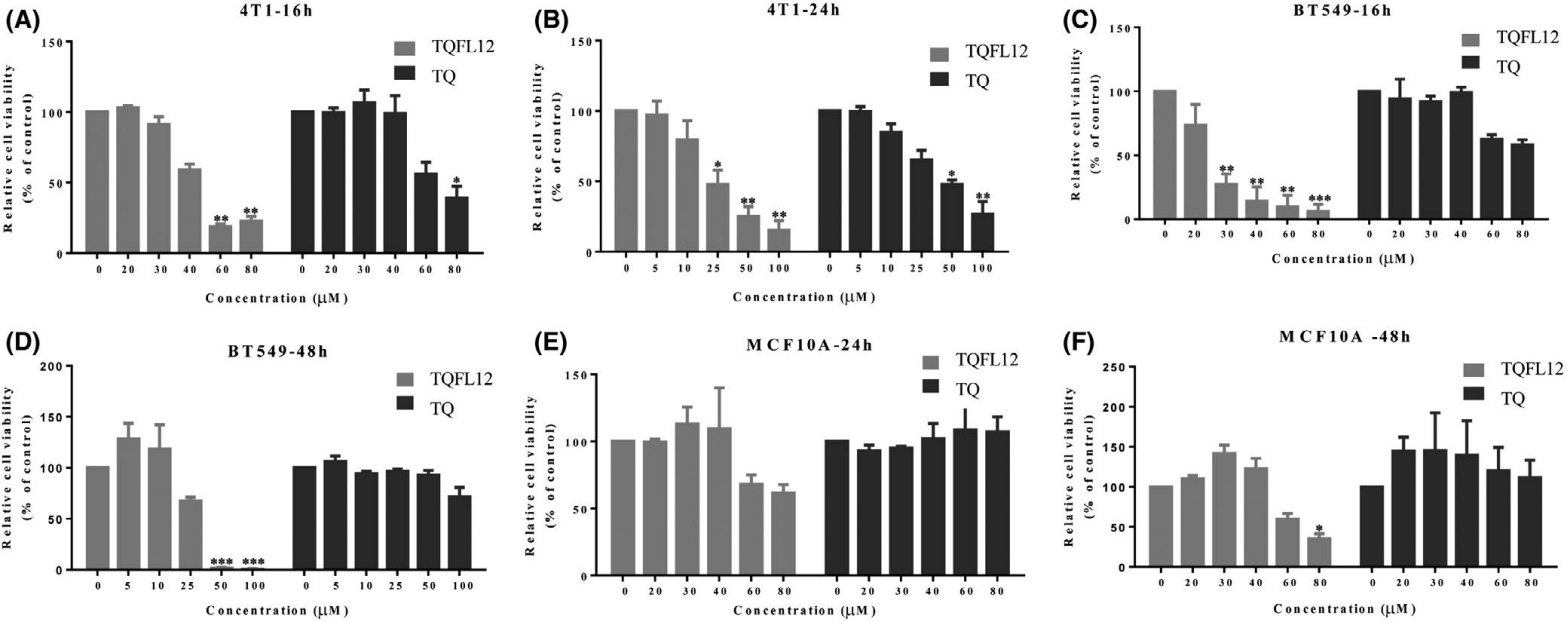
The biological effects of TQFL12 and TQ on the viability of different triple‐negative breast cancer cell lines. A & B. CCK8 assay showed the effect of TQFL12 and TQ on 16 h (A) and 24 h (B) in 4T1 cell line. C & D. The effect of TQFL12 and TQ on 16 h (C) and 48 h (D) in BT549 cell line. E & F. The effect of TQFL12 and TQ on 24h (E) and 48h (F) in normal breast epithelial cell line MCF‐10A. The results are expressed as the mean ± SD (*n* = 3, *p < 0.05, 0.05<**p < 0.001, ***p < 0.001)

To determine whether TQFL12 has displayed specific cytotoxic effect on breast cancer cells than TQ, we performed systemic analyses of the cytotoxic effect of TQFL12 and TQ on cell viabilities of TNBC cell lines. To do so, we treated human breast cancer cells (BT549 and MDA‐MB‐231) and mouse breast cancer cell line (4T1) with TQFL12 and TQ at different time points and measured cell viability by CCK8 assays. As shown in Figure 2A~D and Figure S7, TQFL12’s cytotoxic effects on these cell lines were in a time‐dependent manner. We also found that the cytotoxic sensitivity of TQFL12 is higher than TQ on different TNBC cells. To demonstrate whether the toxic effect of TQFL12 is cancer‐specific, we compared the IC_50_ of the TQFL12 on a normal mammary epithelial cell line (MCF10A). The viability of MCF10A cells are shown in Figure 2E &F and the IC_50_ (Table 1 and Table 2) for MCF10A is significantly higher (>100µM) than that of all TNBC cell lines (<100µM). The IC_50_ of TQ12 against TNBC cells 4T1 was as low as 20.24 μM. These results indicated that the cytotoxic effect of TQFL12 is more sensitive than TQ.

**TABLE 1:** The IC_50_ value of TQFL12 and TQ in 4T1 cell line

Cell type	IC_50_ value at 16 h (μM)		IC_50_ value at 24 h (μM)	
Cell line	TQFL12	TQ	TQFL12	TQ
4T1	43.101	74.038	20.241	45.22

**TABLE 2:** The IC_50_ values of TQFL12 and TQ in breast cancer cell lines

Cell types	IC_50_ values at 16 h (μM)		IC_50_ values at 48 h (μM)	
Cell line	TQFL12	TQ	TQFL12	TQ
BT549	28.729	106.298	27.686	180.679
MDA‐MB−231	29.572	61.688	31.613	107.916
MCF10A	˃100	˃200	74.038	˃200

### 3.3 TQFL12 inhibits breast cancer cell growth, migration and invasion by altering cell apoptosis but slightly affects cell cycle

**FIGURE 3:**
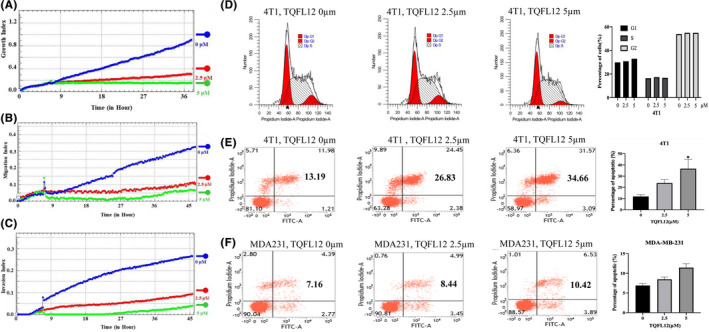
TQFL12 roles in breast cancer cell growth, migration, invasion, cell cycle and apoptosis. A~C. TQFL12 inhibits breast cancer cell growth (A), migration (B) and invasion (C) in the indicated TQFL12 concentration. D. Cell cycle of 4T1 in the indicated TQFL12 concentration. E. Cell apoptosis of 4T1 in the indicated TQFL12 concentration. F. Cell apoptosis of MDA‐MB‐231 (MDA231) in the indicated TQFL12 concentration. ‘*’ indicates *p* < 0.05. *p* value <0.05 was considered different

EMT (epithelial‐to‐mesenchymal transition) is correlated with cancer metastasis. Inhibition of EMT might be a valuable approach in cancer therapy. It was reported that TQ could inhibit breast cancer cell growth, migration and invasion.^16^ To determine the specific effects of TQFL12, we analysed its effect on cancer cell growth, migration and invasion of 4T1 cell using real‐time cell analyser. As such, we examined the effect of different concentrations (0, 2.5, 5 μM) of TQFL12 on 4T1 cell line. The cell index results revealed that TQFL12 significantly inhibits cancer cell growth (Figure 3A), migration (Figure 3B) and invasion (Figure 3C) at 2.5 and 5 μM.

Given the fact that TQFL12 treatment not only suppressed cancer cell migration and invasion but also led to cell death, we next examined the pro‐apoptotic effect of TQFL12 by flow cytometry in 4T1 and MDA‐MB‐231 breast cancer cell lines. The results showed that TQFL12 slightly or little affects the cell cycle (Figure 3D) but significantly affects cell apoptosis in 4T1 cells with 5µm TQFL12 treatment (Figure 3E, *p* < 0.05). In contrast, it had no effect on the cell cycle (data not shown) and minor effects on cell apoptosis in MDA‐MB‐23 cells (Figure 3F), which is similar to reported results.^22^ Altogether, our studies indicate that TQFL12 significantly inhibits breast cancer cell migration and invasion.

### 3.4 Effects of TQFL12 on breast cancer cell–derived xenograft tumours in vivo

**FIGURE 4:**
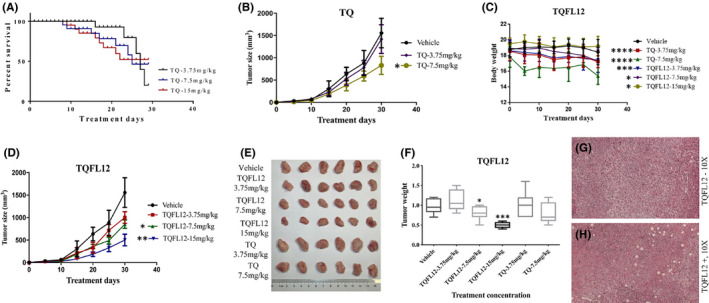
The suppressive effect of TQFL12 on breast cancer growth in an animal model. A. The mouse survival curve of TQ treatment group. B. TQFL12 treatment has no effect on the body weight of the mice, while TQ treatment reduces the body weight of the mice at 7.5 mg/kg. C & D. TQ (C) and TQFL12 (D) suppress tumour growth measured with the tumour size through dose‐dependent manners. E & F. TQ and TQFL12 suppress tumour growth measured with the tumour volumes through dose‐dependent manner; TQFL12 has stronger tumour‐suppressive effect than TQ. G & H. Representative images for *in situ* tumours without (G) and with (H) TQFL12 treatments (7.5 mg/kg). ‘*’ indicates *p* < 0.05, ‘**’ indicates *p *< 0.01. *p* value <0.05 was considered different, *p* value <0.01 was considered significantly different

To demonstrate that TQFL12 is capable of repressing breast cancer cell growth, migration and invasion *in vivo*, we conducted a series of experiments with the congenic mouse tumour xenograft model. To do so, we first established the animal model of breast cancer by injecting 4T1 cells into the 4th mammary fat pads of BALB/c female mice. Four days after injection of the cells, the mice were randomly divided into eight groups with six in each group. The animals were treated with either TQFL12 (0, 3.75, 7.5 and 15 mg/kg) or TQ (0, 3.75, 7.5 and 15 mg/kg) 3 days after injection, and the treatment was boosted every 5 days. The body weights and tumour sizes were measured every 5 days after 4T1 injection. The mice were killed at the end of the 30‐day treatment, and the size and weight of the tumours were recorded. Figure 4A showed the per cent survival of mice in the TQ group. High‐concentration TQ (15 mg/kg) was very toxic, and the mice began to die when the experiment was performed at the 7th day and all died at the 21st day. Figure 4B showed that the TQ treatment reduced tumour size. Meanwhile, the body weights of the animals showed that the toxic effect were more dramatic in the treatment of TQ than that in TQFL12 treatment (Figure 4C). As shown in Figure 4E‐H that compared to control, the sizes, weights and tissue morphology of the tumours were significantly reduced by TQFL12 and TQ treatment in a dose‐dependent manner. Importantly, TQFL12 is less toxic compared with TQ (Figure 4E‐F). The statistical analysis for comparing between the groups of TQFL12 (7.5mg/kg) and TQ (7.5mg/kg) are presented in Figure S8.

### 3.5 TQFL12 inhibits breast cancer cell invasion and migration better than TQ in vivo

**FIGURE 5:**
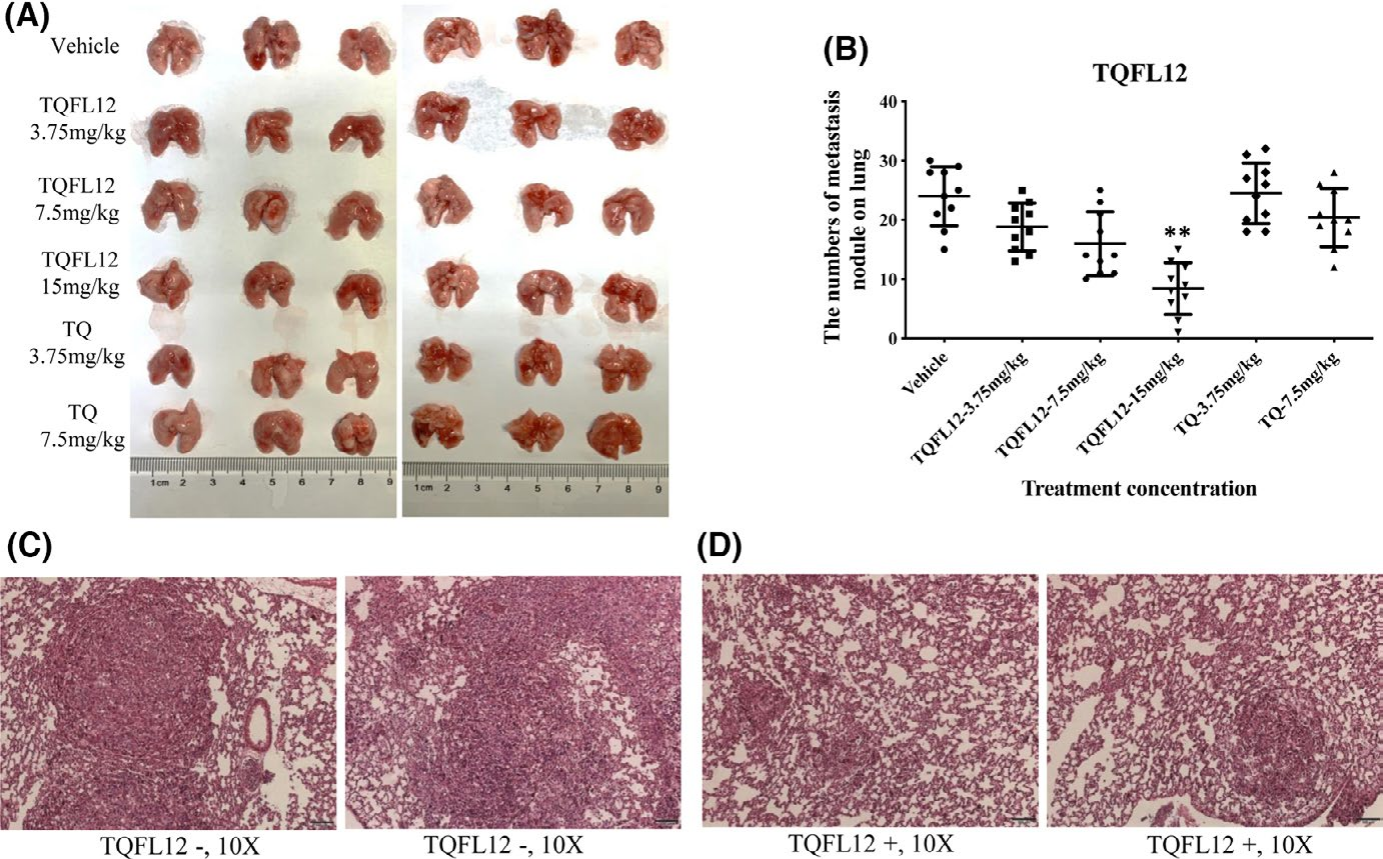
TQFL12 treatment inhibits breast cancer metastasis to lung. A. The size of metastasis colonies on lung in TQFL12 treatment group was smaller than those in TQ treatment group. B. The numbers of metastasis nodule on lung in TQFL12 treatment group was less than in TQ treatment group. C. Representative images for metastatic tumours in lungs without TQFL12 treatments. D. Representative images for metastatic tumours in lungs with TQFL12 treatments

**FIGURE 6:**
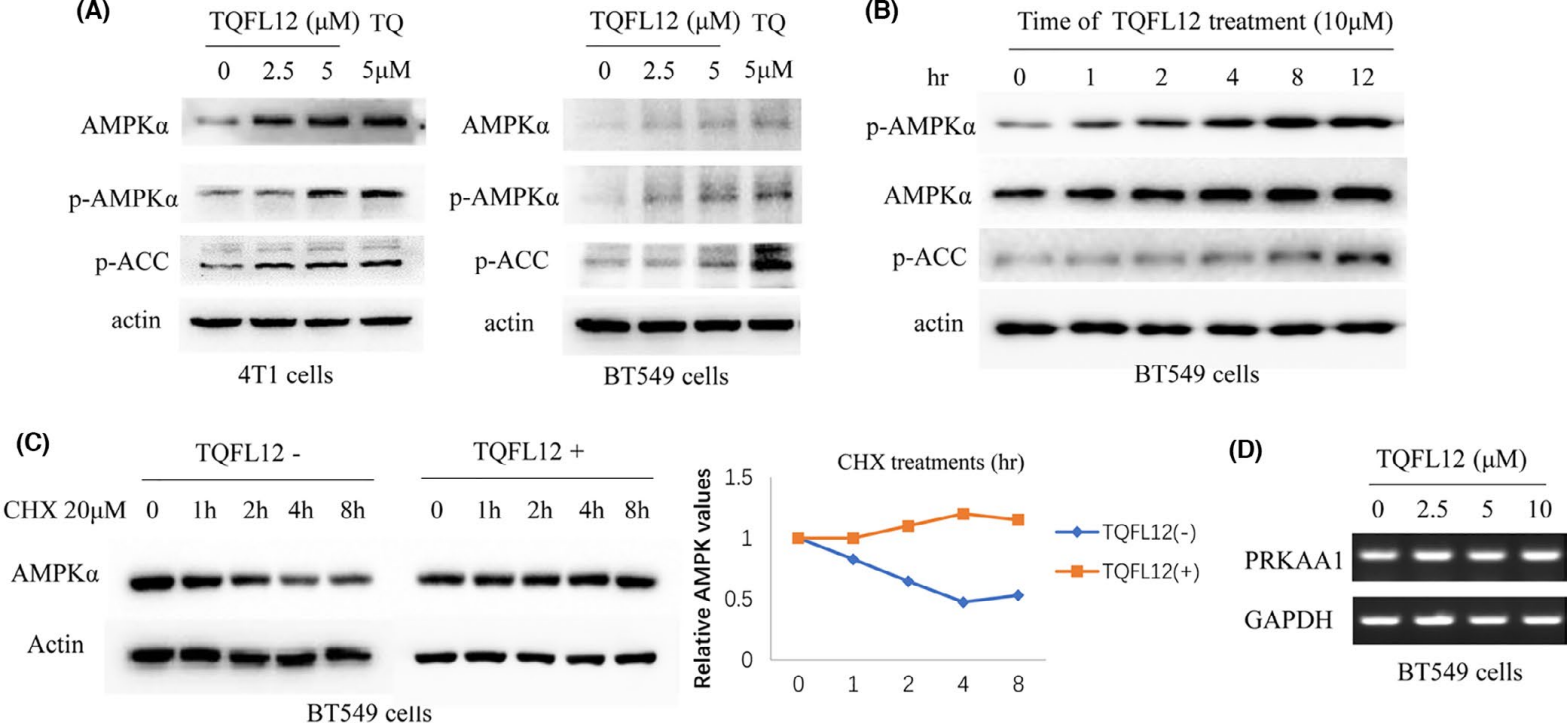
TQFL12 effects AMPKα signalling in breast cancer cell. A. Total and phosphorylated protein levels of AMPKα; B. time courses on total and phosphorylated protein levels of AMPKα; C. the treatments for cycloheximide (CHX) and TQFL12. Left panel, Western blot with indicated antibodies and indicated CHX time hours (hr); right panel, quantitative results of left panel; D. Results of semi‐quantitative RT‐PCR for mRNA of *PRKAA1*

Using breast cancer cell–derived xenograft model, we also investigated the effects of TQFL12 treatment on tumour cell migration/invasion as estimated by the number of colonies in the lungs. Figure 5A showed numerous colonies formed in the lungs of the vehicle‐treated group, whereas only a few colonies were identified in the TQFL12‐treated mice. In addition, the overall sizes of the colonies in the control group are significantly bigger than those in the TQFL12‐treated mice. Furthermore, Figure 5B showed that the average number of colonies per mice, which represents cancer cell migration and invasion, reduced in a dose‐dependent manner when the mice were treated with TQ. Meanwhile, compared to TQFL12‐treated group, there are more and larger colonies in the lungs of the TQ‐treated group on the same concentration (Figure 5CandD). Interestingly, images of *in situ* tumours treated with TQFL12 showed more air bubbles compared to those of the control group (Figure 6E, right panel via left panel). These *in vivo* data unambiguously demonstrated that TQFL12 is better capable at inhibiting breast cancer cell growth, migration and invasion.

### 3.6 TQFL12 affects AMPK signalling and stabilizes itself in triple‐negative breast cancer cells

We next sought to explore the underlying signalling pathways that are affected by which TQFL12 controls tumour progression. Therefore, we performed Western blot using different cancer cell lines treated with TQFL12. Results showed that, similar to TQ,^29^ TQFL12 affects AMPKα (5'‐adenosine monophosphate–activated protein kinase alpha) total and phosphorylated protein levels in both 4T1 cells (Figure 6A, left panel) and BT549 (Figure 6B, right panel). Accordingly, p‑ACC (phosphorylated acetyl‑CoA carboxylase), an AMPKα down‐stream target, was also up‐regulated (Figure 6A).

Then, timing of TQFL12 treatment was executed and found that, comparing to the 0 hour (no TQFL12 treatment), total and phosphorylated AMPKα protein levels are gradually increased from 1 h, and at 8 h, reaches a peak (Figure 6B). Accordingly, p‑ACC was also gradually increased from 1 h and, at 12 h, reaches a peak (Figure 6B). These results indicated that TQFL12 may directly affect AMPKα protein stability.

Then, we tested whether TQFL12 stabilizes AMPK. To do so, BT549 cells were treated with or without TQFL12 for 8h, and CHX, an inhibitor protein synthesis, was added with different time points. Cells were harvested and proteins in the lysate were used for Western blotting and band intensities were semi‐quantified. Figure 6C shows the TQFL12 treatments significantly increase AMPKα protein levels (Figure 6C, left panel, Western blots; right panel, quantitative curves). But the mRNA levels for *PRKAA1* showed no difference (Figure 6D), indicating that TQFL12 increase AMPKα protein levels is not due to the increase of mRNA transcription. Altogether, we confirmed that TQFL12 affects AMPK signalling by directly stabilizing AMPKα.

### 3.7 TQFL12 interacts with hydrophobic surface of AMPKα

**FIGURE 7:**
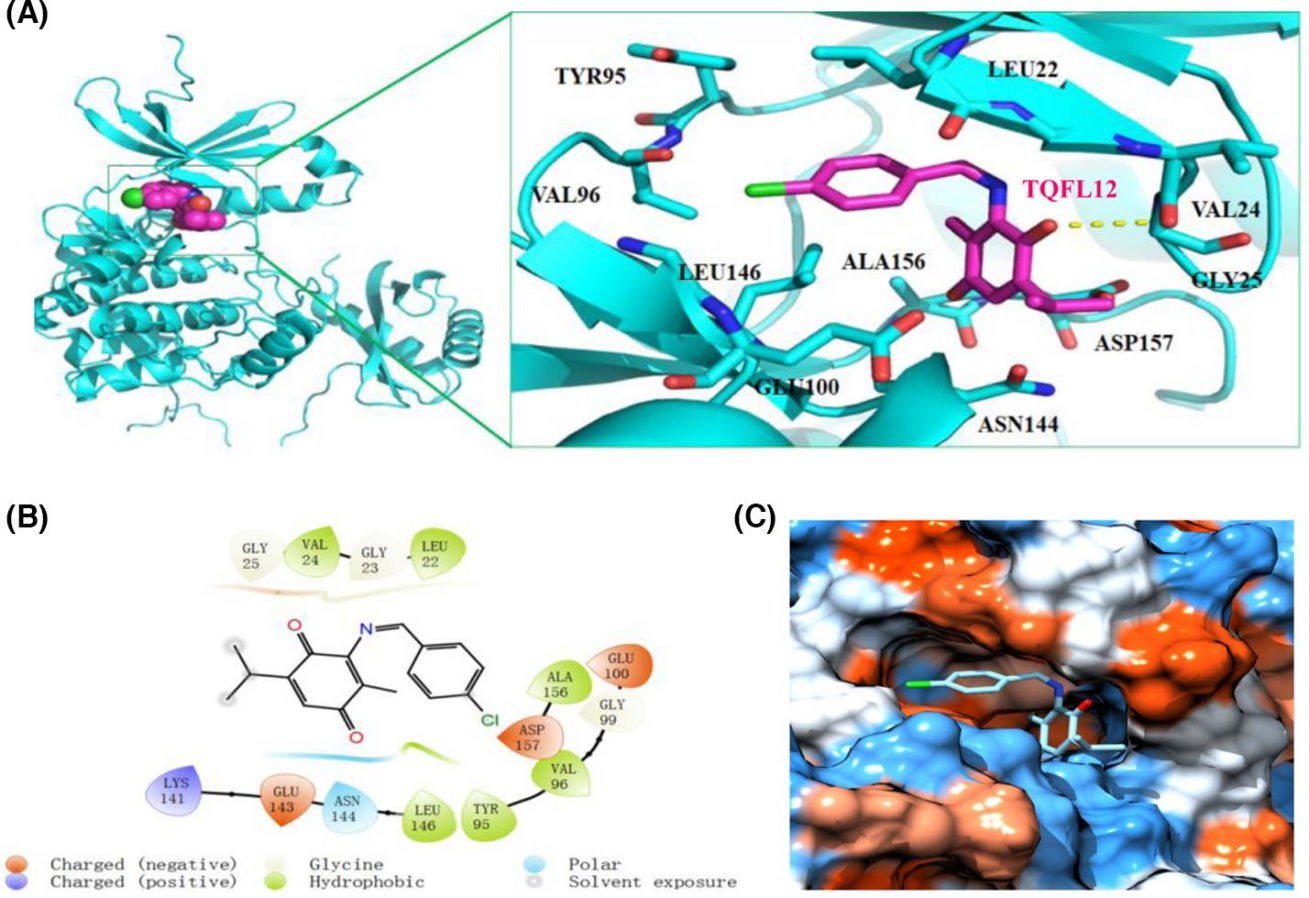
Docking results of compound TQFL12 and AMPKα protein. A. The binding model of TQFL12 and AMPKα; B. The 2D mode of TQFL12 and AMPKα interaction; C. hydrophobic surface of TQFL12 on AMPKα

To further determine whether TQFL12 interacts with AMPKα, molecular docking experiment was conducted. We found that molecular docking score of TQFL12 and AMPKα is −5.08 kcal/mol. TQFL12 can form a strong hydrogen bond with AMPKα’s side chain hydroxy group residue Val24 with the distance of 3.2 Å (Figure 7A). In addition, the benzene ring of TQFL12 can form significant hydrophobic interactions with residue Leu22 (Figure 7B). Furthermore, 2D modelling showed interaction between TQFL12 and AMPKα (Figure 7C), whereas Figure 7C revealed hydrophobic surface of TQFL12 on AMPKα.

## 4 DISCUSSION

TQ is known as an anti‐tumour candidate compound that is characterized by its small molecular size, and recent studies have touched on its biological function and mechanism of action.^20^, ^30^, ^31^, ^32^ While the mechanism and targets of TQ have become the focus of current research, the compound itself fails to exhibited strong anti‐tumour effects in numerous cancer ‐cell studies, which limited the potential of TQ in clinical drug development.^33^ Katharina EN et al.^34^ synthesized several TQ‐substituted compounds by click chemistry reaction, but the synthesized compounds did not show better therapeutic effect than TQ. Sandra R et al^35^ synthesized several derivatives which showed the compounds with unsaturated side chains conferred a greater activity than those compounds with equally long saturated chains, but the *in vivo* efficacy was not evaluated. Other than these, no additional chemical modification of TQ has been reported.^33^, ^36^ Previous studies showed that some synthetic compounds with chlorophenyl exhibited good anti‐cancer activity and it also possessed the potential to improve the bioavailability. We conduct a study of structural modification of TQ by chloromethylation to determine whether there is any benefit of chlorobenzyl modification on TQ. A candidate named TQFL12 or CTQ [(E)‐3‐((4‐chlorobenzylidene) amino)‐5‐isopropyl‐2‐methylcyclohexa‐2, 5‐diene‐1,4‐dione] was found by a series of screenings and exhibited a greater activity than TQ in both *in vitro* and *in vivo* studies.

TNBC is an aggressive and a highly heterogeneous subtype of breast cancer and associated with poor prognosis; it continues to be a leading cause of cancer‐related death in women. Given the TQ’s potentially beneficial effect on human health and breast cancer patients, we speculate its derivative TQFL12 would also possess a therapeutic effect against TNBC. Thus, we first performed a systematic analysis of TQFL12 and TQ by CCK8 assay on TNBC cells, and the result showed that TQFL12 is more toxic to breast cancer cells than normal cells, indicating that TQFL12 is more effective than TQ on tumour cytotoxicity. Further studies revealed that TQFL12 significantly inhibited cancer cell growth, invasion and migration in a dose‐dependent manner both *in vitro* and *in vivo*.

Epithelial‐to‐mesenchymal transition (EMT) and EMT‐transcriptional factors (TFs) are correlated with cancer metastasis, and our previous study indicated TQ inhibited TWIST1 and EMT‐related makers.^16^ Inhibition of EMT may be a mechanism of TQFL12. Thus, we tested most EMT‐related proteins and TFs, including E‐Cadherine, N‐Cadherine, Vimentin, Claudin‐1/3, TWIST1, Snail1, but no significant changes were found in some of TNBC cell lines (data not shown). Further mechanistic study and molecular docking showed that TQFL12 directly targets and stabilizes AMPKα protein, thus inhibiting TNBC metastasis.

AMPK, a protein kinase, is a cellular energy sensor with key roles in regulating energy haemostasis and cellular metabolism, including fatty acid and cholesterol synthesis by ACC (phosphorylating acetyl‐CoA carboxylase) and LIPE (hormone‐sensitive lipase) enzymes, respectively.^37^, ^38^ ACC, the rate‐limiting step in fatty acid synthesis, is a biotin‐containing enzyme that catalyses the carboxylation of acetyl‐CoA to malonyl‐CoA.^39^ AMPK may inhibit tumorigenesis and metastasis via regulating cell growth, proliferation, autophagy, ferroptosis, stress responses and cell polarity.^25^, ^40^, ^41^, ^42^ Thus, AMPK could be an important target for cancer therapeutics.^42^, ^43^ Herb‐derived medication is considered as one of the main activators of AMPK, and surprisingly, our results indicate that it could be a better target for the TQ derivative TQFL12. Taken together, our discovery of TQFL12’s effect on the AMPK pathway in cancer is significant and could have great clinical potential.

## 5 CONCLUSION

In summary, by conducting systematic and unbiased analyses of TQFL12’s effects on TNBC, we found that TNBC cells are more sensitive to TQFL12 treatment compared with TQ treatment, and TQFL12 affects cancer cell migration and invasion both *in vitro* and *in vivo*. We speculate that TQFL12 could have great potential as an option of adjunct targeted therapies as for breast cancer patients. Moreover, mechanism study indicates that TQFL12 increases AMPK/ACC activity by stabilizing AMPK protein. Altogether, our findings suggest that TQFL12, as a novel TQ derivative, has a higher anti‐tumour effect on breast cancer cells with less toxic effect. Thus, it could be a candidate compound for the treatment of TNBC and have potential clinical values.

## CONFLICT OF INTEREST

No.

## AUTHOR CONTRIBUTIONS

**Chunli Wei:** Data curation (equal); Investigation (equal); Validation (equal); Writing‐original draft (equal); Writing‐review & editing (equal). **Hui Zou:** Investigation (equal); Resources (equal); Writing‐review & editing (equal). **Ting Xiao:** Investigation (equal). **Xiaoyan Liu:** Investigation (equal). **Qianqian Wang:** Validation (equal). **Jingliang Cheng:** Investigation (equal). **Shangyi Fu:** Writing‐original draft (equal); Writing‐review & editing (equal). **Xin Xie:** Supervision (equal). **Jiangzhou Peng:** Supervision (equal). **JUNJIANG FU:** Conceptualization (equal); Funding acquisition (equal); Project administration (equal); Resources (equal); Supervision (equal); Writing‐original draft (equal); Writing‐review & editing (equal).

## Supporting information

Fig S1‐S8

## Data Availability

Data sharing not applicable to this article as no datasets were generated or analysed during the current study.
