# Supplementary material for: TQFL12, a novel synthetic derivative of TQ, inhibits triple‐negative breast cancer metastasis and invasion through activating AMPK/ACC pathway
Source: J Cell Mol Med. 2021 Oct 5;25(21):10101–10. doi: 10.1111/jcmm.16945 (PMC8572774; doi:10.1111/jcmm.16945)

Supplementary information

Supplementary **Figure 1.** ^{1}H NMR spectrum of the novel compound NTQ.

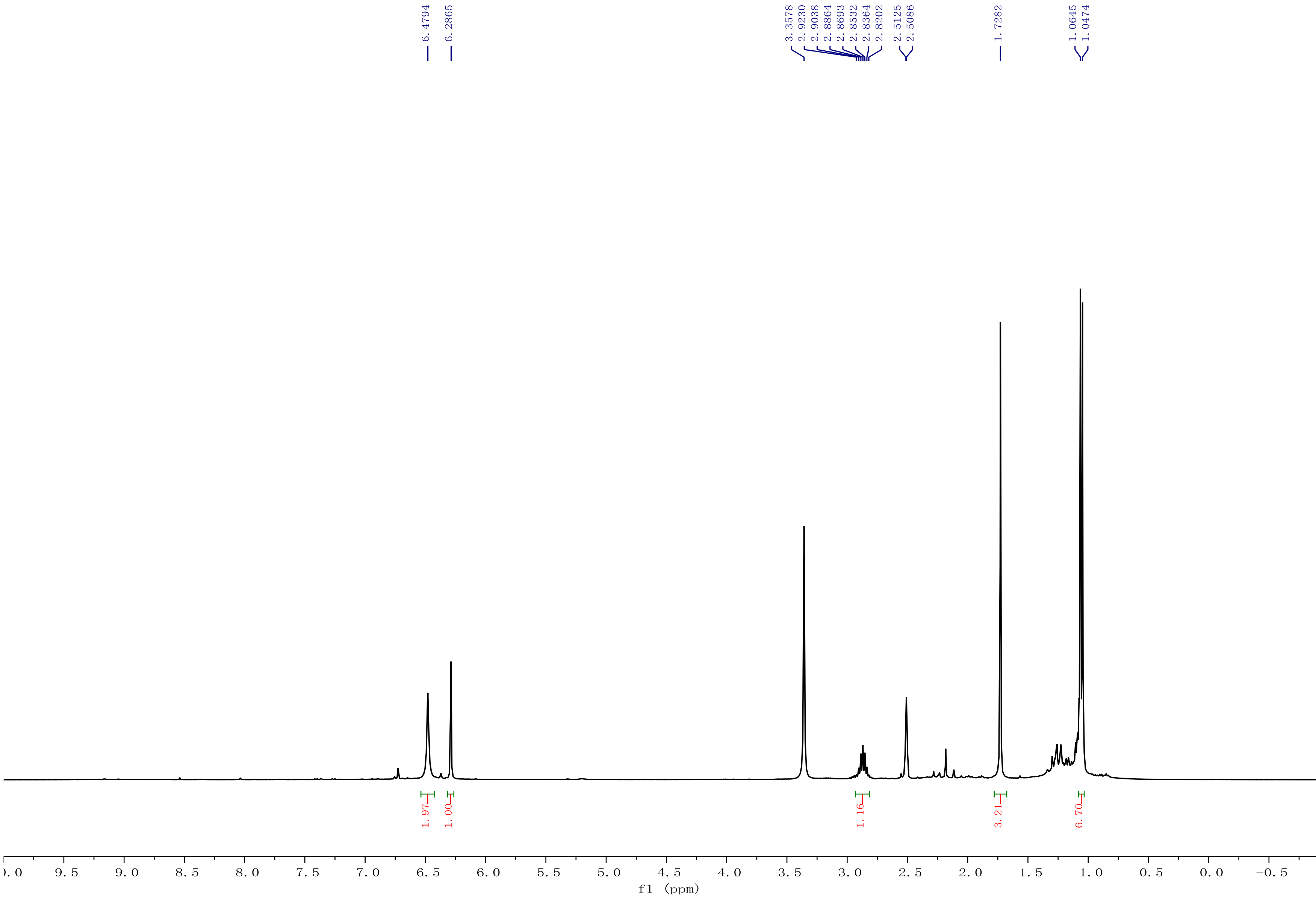

Supplementary **Figure 2.** ^{13}C NMR spectrum of the novel compound NTQ.

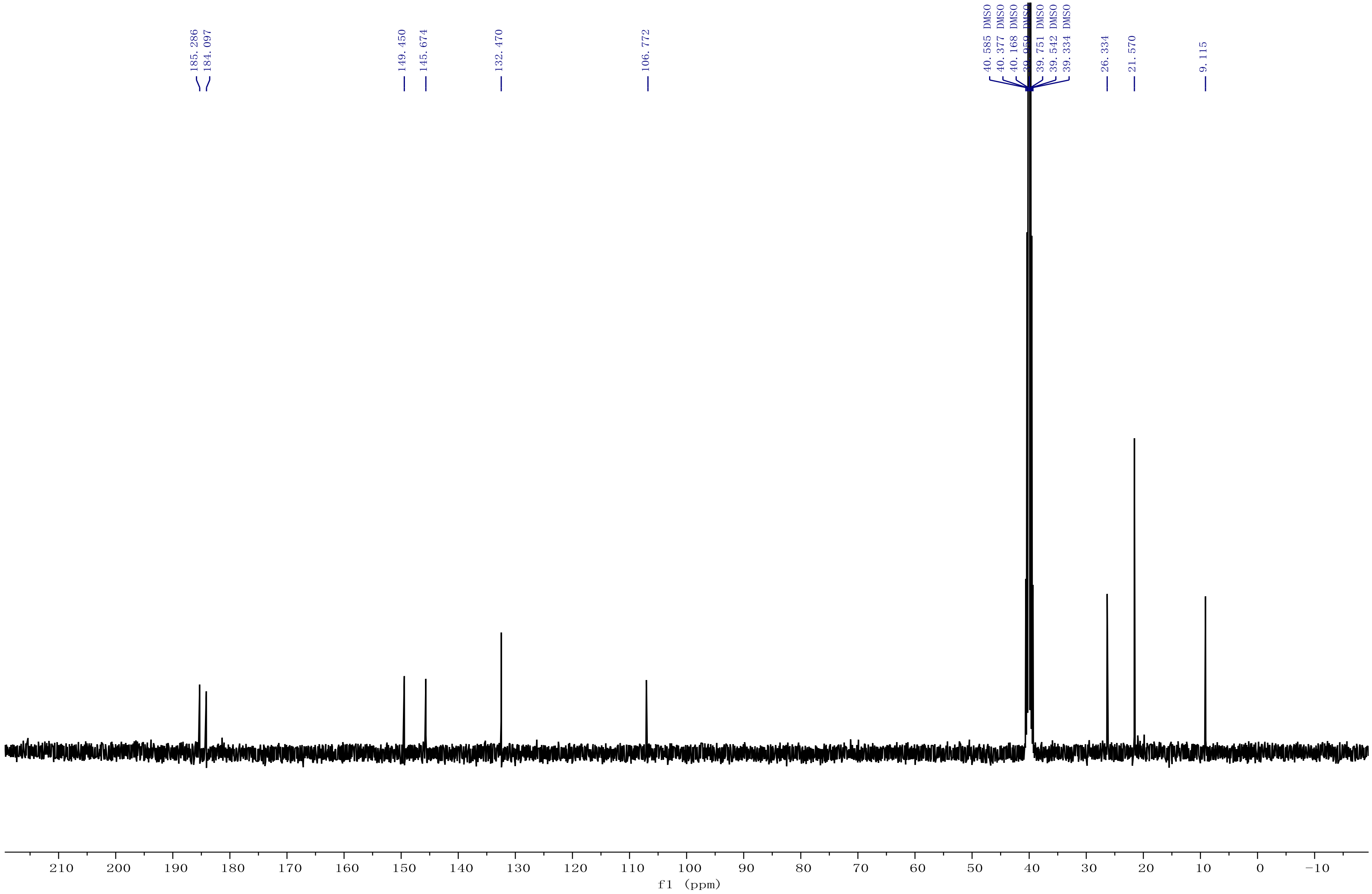

Supplementary **Figure 3.** ^{1}H NMR spectrum of the novel compound TQFL12.

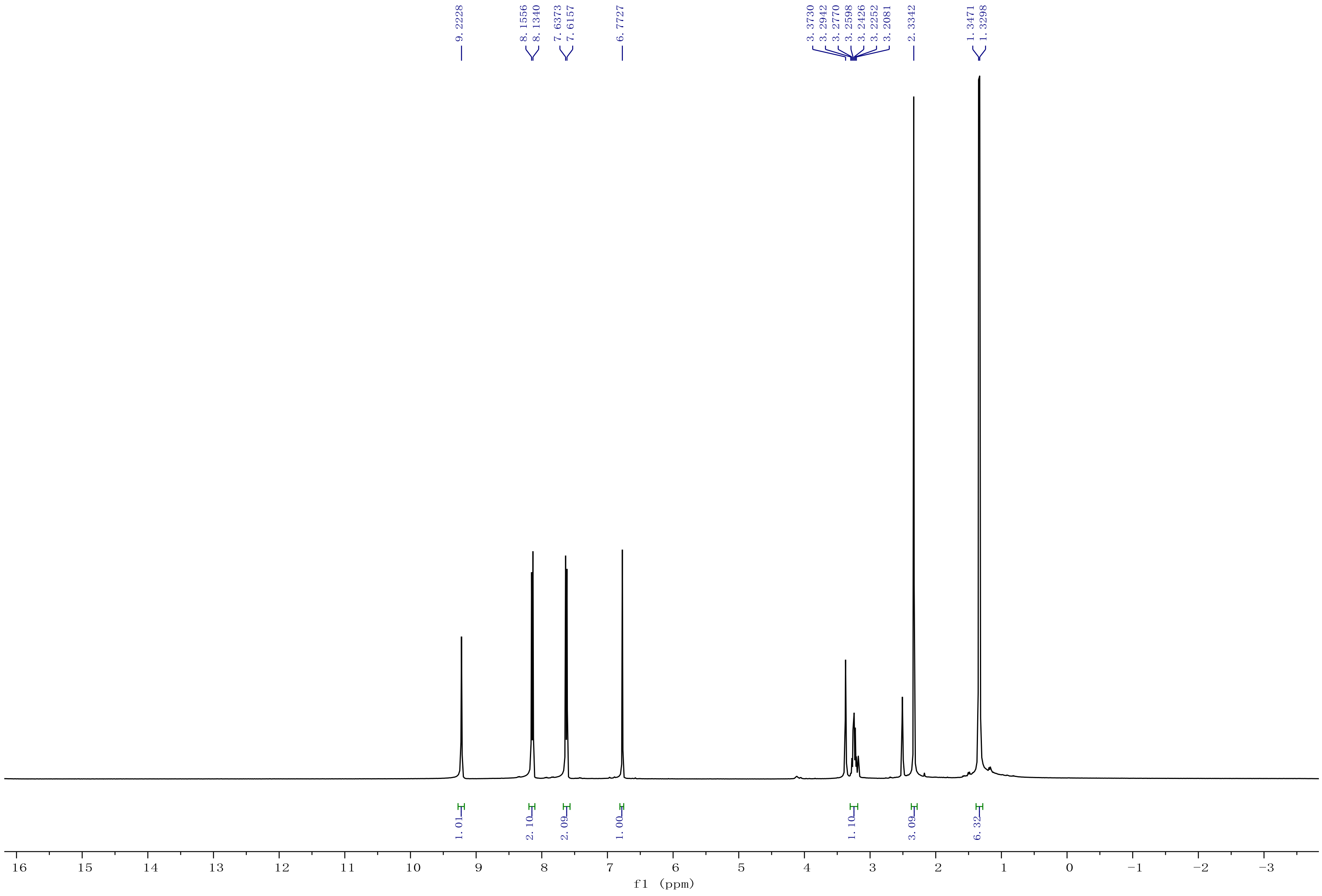

Supplementary **Figure 4.** ^{13}C NMR spectrum of the novel compound TQFL12.

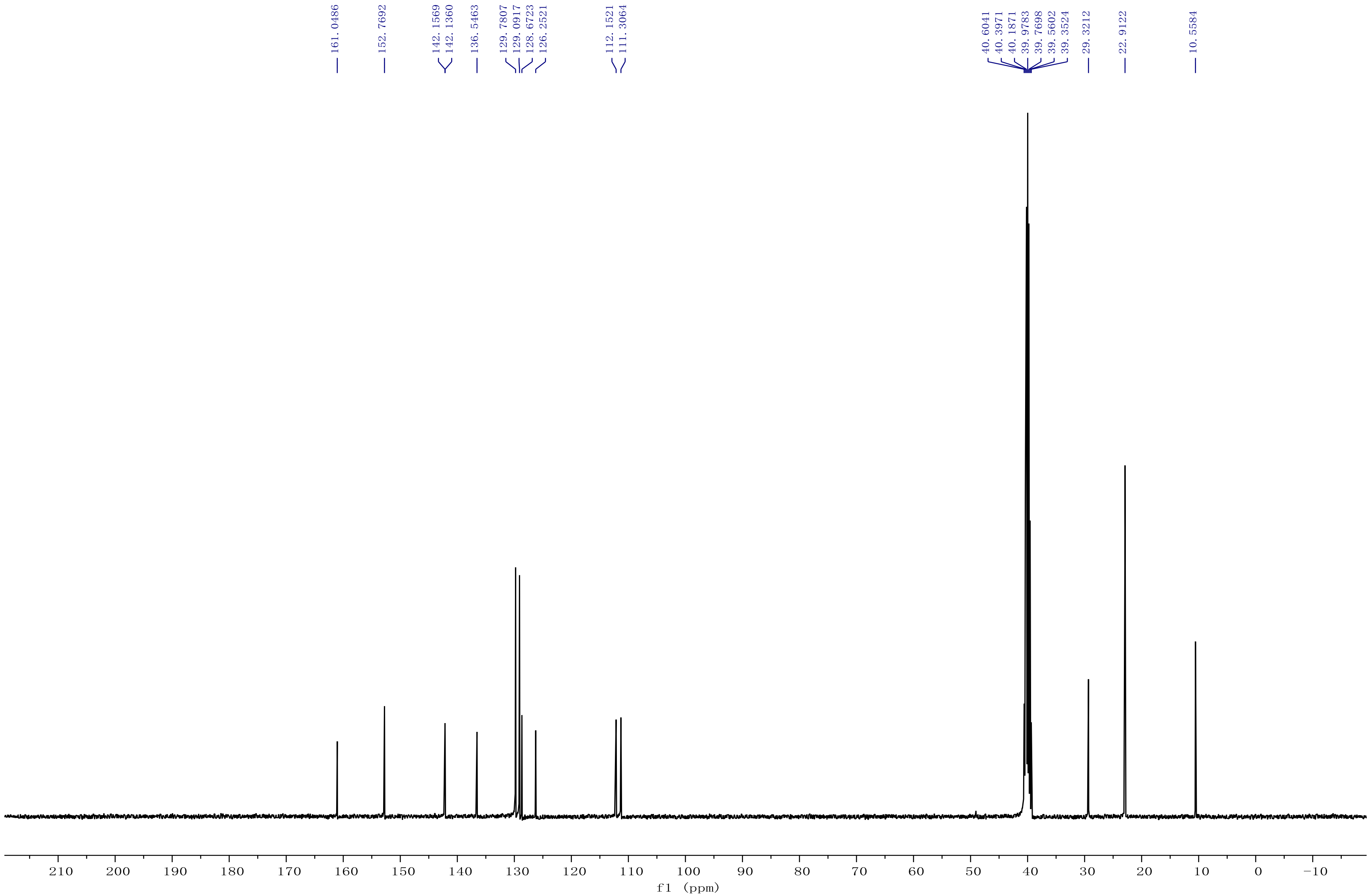

Supplementary **Figure 5.** HR-ESI-MS spectrum of the novel compound TQFL12.

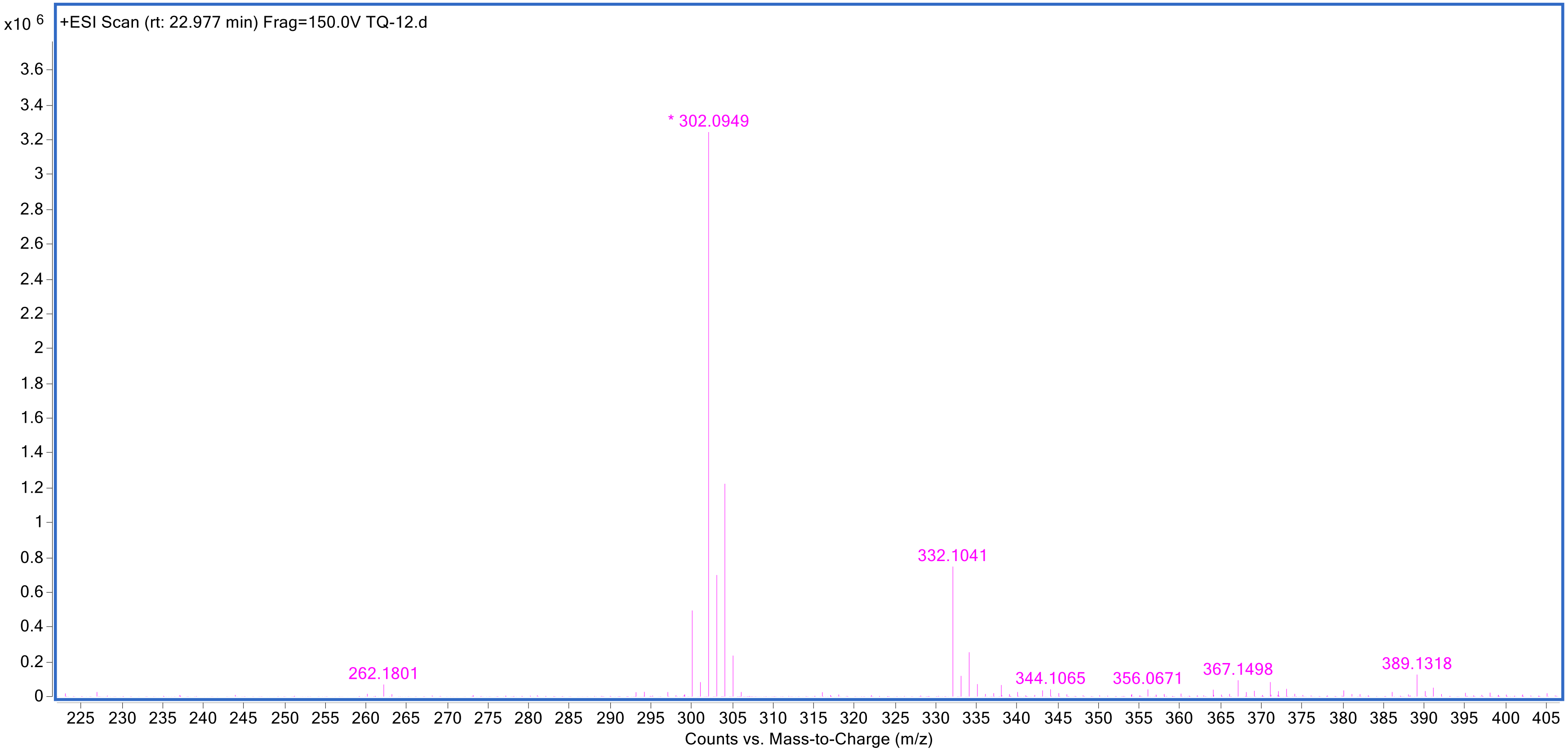

Supplementary **Figure 6.** The purity of the compound TQFL12 was >98.2%.

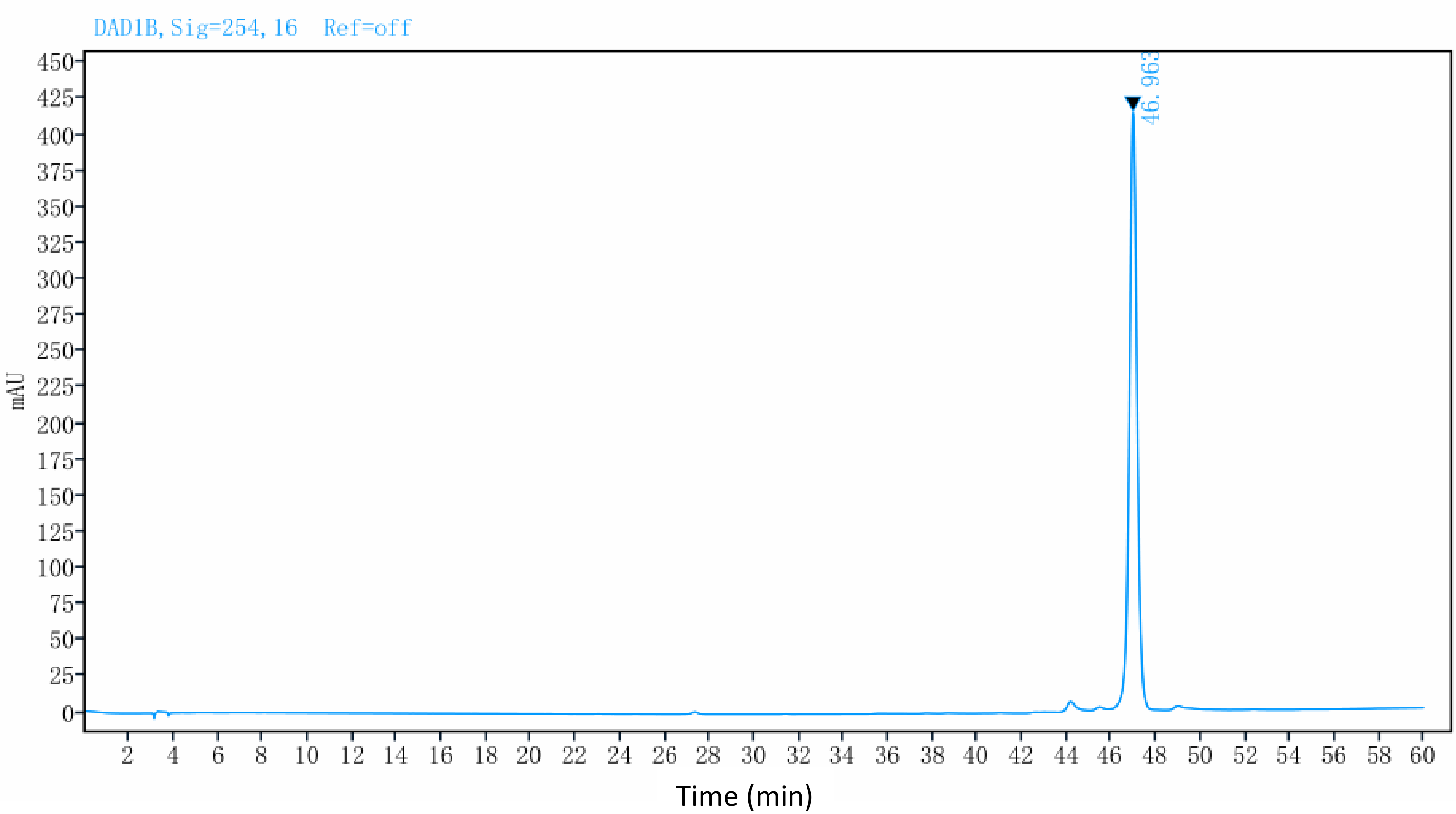

Signals: DAD1B, Sig=254, 16 Ref=off

Sample Components	Retention time [min]	Peak area	Percentage of peak area	Peak height	Peak width	Resolution	Theoretical plates USP
1	46.96	9850.992	98.20	413.743	2.500	4.39016	95035.78120

Supplementary **Figure 7.** The biological effects of TQFL12 and TQ on the viability of triple-negative breast cancer cell line MDA-MB-231 BY CCK8 assays for TQ-12 in MDA-MB-231 cells. Left panel, 16 hours treatments; right panel, 48 hours treatments

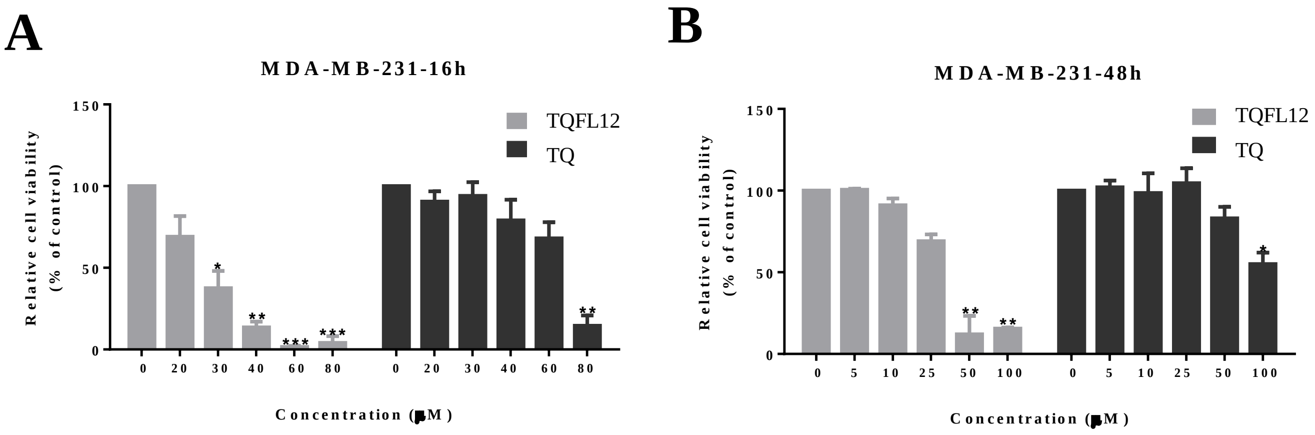

Supplementary **Figure 8.** The statistic analysis for comparing between the groups of TQFl12(7.5mg/kg) and TQ(7.5mg/kg). A. Body weight. B. Tumor weight. C. Tumor size. D. Metastasis on the lungs.

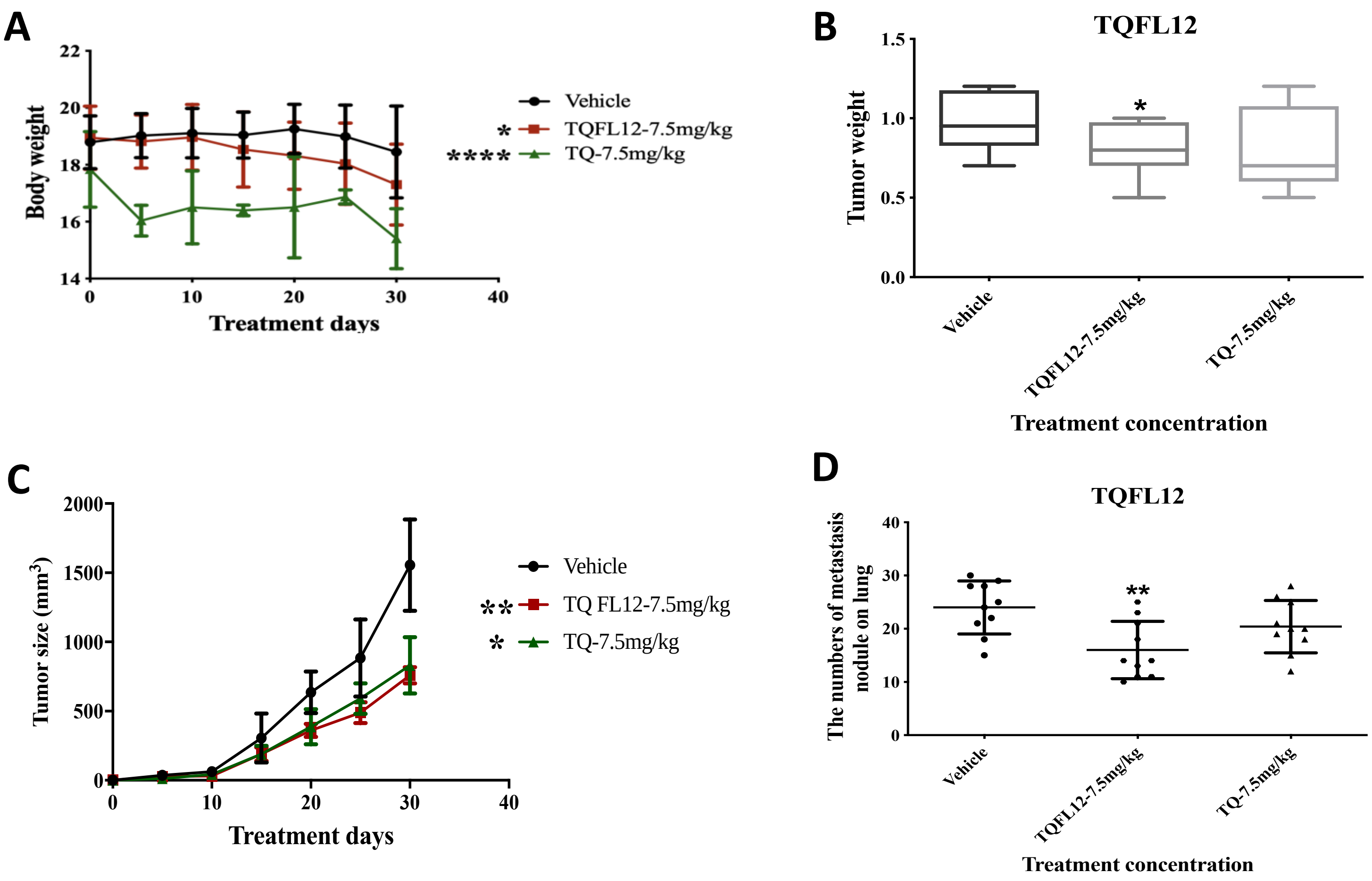

Supplement: Supplementary file 1 — Fig S1‐S8 [file JCMM-25-10101-s001.pdf]
